# Bacteriophage treatment is effective against carbapenem-resistant *Klebsiella pneumoniae* (KPC) in a neutropenic murine model of gastrointestinal translocation and renal infection

**DOI:** 10.1128/aac.00919-24

**Published:** 2024-12-20

**Authors:** Panagiotis Zagaliotis, Jordyn Michalik-Provasek, Eleftheria Mavridou, Ethan Naing, Ioannis S. Vizirianakis, Dimitrios Chatzidimitriou, Jason J. Gill, Thomas J. Walsh

**Affiliations:** 1 Transplantation/Oncology Program, Division of Infectious Diseases, Weill Cornell Medicine 639780 , New York, New York, USA; 2 Department of Pharmacology, School of Pharmacy, Aristotle University of Thessaloniki 82957 , Thessaloniki, Greece; 3 Department of Microbial Pathogenesis and Immunology, Texas A&M University 14736 , College Station, Texas, USA; 4 Department of Health Sciences, School of Life and Health Sciences, University of Nicosia 121343 , Nicosia, Cyprus; 5 Deparment of Microbiology, School of Medicine, Aristotle University of Thessaloniki 37783 , Thessaloniki, Greece; 6 Department of Animal Science, Texas A&M University Department of Animal Science 199048 , College Station, Texas, USA; 7 Center for Phage Technology, Texas A&M University 14736 , College Station, Texas, USA; 8 Center for Innovative Therapeutics and Diagnostics, Richmond, Virginia, USA; Johns Hopkins University School of Medicine, Baltimore, Maryland, USA

**Keywords:** carbapenem-resistant (KPC), *Klebsiella pneumoniae* (KPC), bacteriophages, neutropenic murine model, gastrointestinal translocation, renal infection

## Abstract

Carbapenemase-producing *Klebsiella pneumoniae* (KPC) are globally emerging pathogens that cause life-threatening infections. Novel treatment alternatives are urgently needed. We therefore investigated the effectiveness of three novel bacteriophages (Spivey, Pharr, and Soft) in a neutropenic murine model of KPC gastrointestinal colonization, translocation, and disseminated infection. Bacteriophage efficacy was determined by residual bacterial burden of KPC (CFU/g) in kidneys. Parallel studies were conducted of bacteriophage pharmacokinetics and resistance. Treatment of mice with 5 × 10^9^ PFU of phage cocktail via intraperitoneal injection was effective in significantly reducing renal KPC CFU by 100-fold (*P* < 0.01) when administered every 24 h and 1000-fold (*P* < 0.01) every 12 h. Moreover, a combination of bacteriophage and ceftazidime-avibactam produced a synergistic effect, resulting in a 10^5^-fold reduction in bacterial burden in cecum and kidney (*P* < 0.001 in both tissues). Prophylactic administration of bacteriophages via oral gavage did not prevent KPC translocation to the kidneys. Bacteriophage decay determined by linear regression of the ln of mean concentrations demonstrated R^2^ values in plasma of 0.941, kidney 0.976, and cecum 0.918, with half-lives of t_1/2_ = 2.5 h. Furthermore, a phage-resistant mutant displayed increased sensitivity to serum killing *in vitro*, but did not show significant defects in renal infection *in vivo*. A combination of bacteriophages demonstrated significant efficacy alone and synergy with ceftazidime/avibactam in the treatment of experimental disseminated KPC infection in neutropenic mice.

## INTRODUCTION

Carbapenemase-producing *Klebsiella pneumoniae* (KPC) is a highly antibiotic-resistant pathogen, which is responsible for nosocomial infections worldwide (1–3). KPC has become endemic in large parts of the world, including numerous metropolitan areas, Greece, and Israel. The global expansion of KPC is largely attributed to the spread of the sequence type (ST) 258 strain, which emerged over the past three decades (4–6). KPC encodes the *bla_KPC_* gene, which produces the carbapenemases. *Bla_KPC_* is located within a Tn3-type transposon, which facilitates plasmid-mediated horizontal transfer, and is therefore easily transferred horizontally, thus spreading resistance within the species, as well as from one geographical location to another (7). Efforts to address these clinical challenges include enhanced infection control practices, better screening methods, optimal usage of existing antibiotics, and development of novel antimicrobial agents (8). ST258, which was studied herein and encodes KPC-2, constitutes approximately 80% of all KPC isolates (9), thereby posing a major threat in hospital settings, especially for critically ill patients in the intensive care unit and immunocompromised patients (10, 11).

There is an increasing demand for novel therapeutic strategies and alternatives (12–14). One promising alternative to antibiotics is bacteriophage therapy, which uses bacteria-killing viruses first discovered in 1917 (15). The discovery and widespread use of antibiotics in the second half of the 20th century overshadowed phage treatments because they were more complex to produce, labor-intensive, time-consuming to prepare, and challenging in administering standardized doses. Yet, with the emergence of bacteria resistance, there has been a resurgence of interest in this therapy, which has shown optimal outcomes against various infections in both preclinical and clinical settings (16–18).

Understanding the pathogenesis of KPC infections is essential to developing novel therapeutics against these diseases. KPC mainly colonizes the alimentary tract, and many epidemiological studies have shown that most types of KPC infection are preceded by gastrointestinal colonization (12–14, 19). Neutrophils play an important role in innate immunity and in gastrointestinal host defense against invasive pathogenic bacteria (20). Disruption of gastrointestinal mucosa by cytotoxic chemotherapy in patients with cancer permits translocation of bacteria into submucosal tissue, into the bloodstream, and subsequently into deep tissues, including the kidneys. Neutrophils normally phagocytose and destroy bacterial cells within the submucosal tissue and in the systemic circulation; however, neutropenia permits unchecked submucosal proliferation and hematogenous invasion into tissue capillaries with subsequent systemic dissemination. We therefore studied the effect of bacteriophages on gastrointestinal translocation and hematogenous dissemination by KPC during cyclophosphamide-induced neutropenia.

Bacteriophages have been used in clinical settings alone or in combination with antibacterial agents for the treatment of refractory bacterial infections with a relatively high success rate (9–12). While preclinical models of bacteriophage treatment of KPC infection also have been studied (16–18, 21), there have been no clinical studies to our knowledge of bacteriophages in the treatment of serious infections caused by KPC in profoundly immunocompromised patients, where attributable mortality may be especially severe (13, 14). In order to achieve a better preclinical understanding of the potential efficacy of bacteriophage against KPC in this patient population, we conducted an investigation of bacteriophages in the treatment of gastrointestinal and disseminated KPC infection in a neutropenic murine model and further studied their interaction with ceftazidime/avibactam (CAZ/AVI).

## RESULTS

### Efficacy of bacteriophages for the treatment of KPC infection of kidneys in neutropenic mice

**FIG 1:**
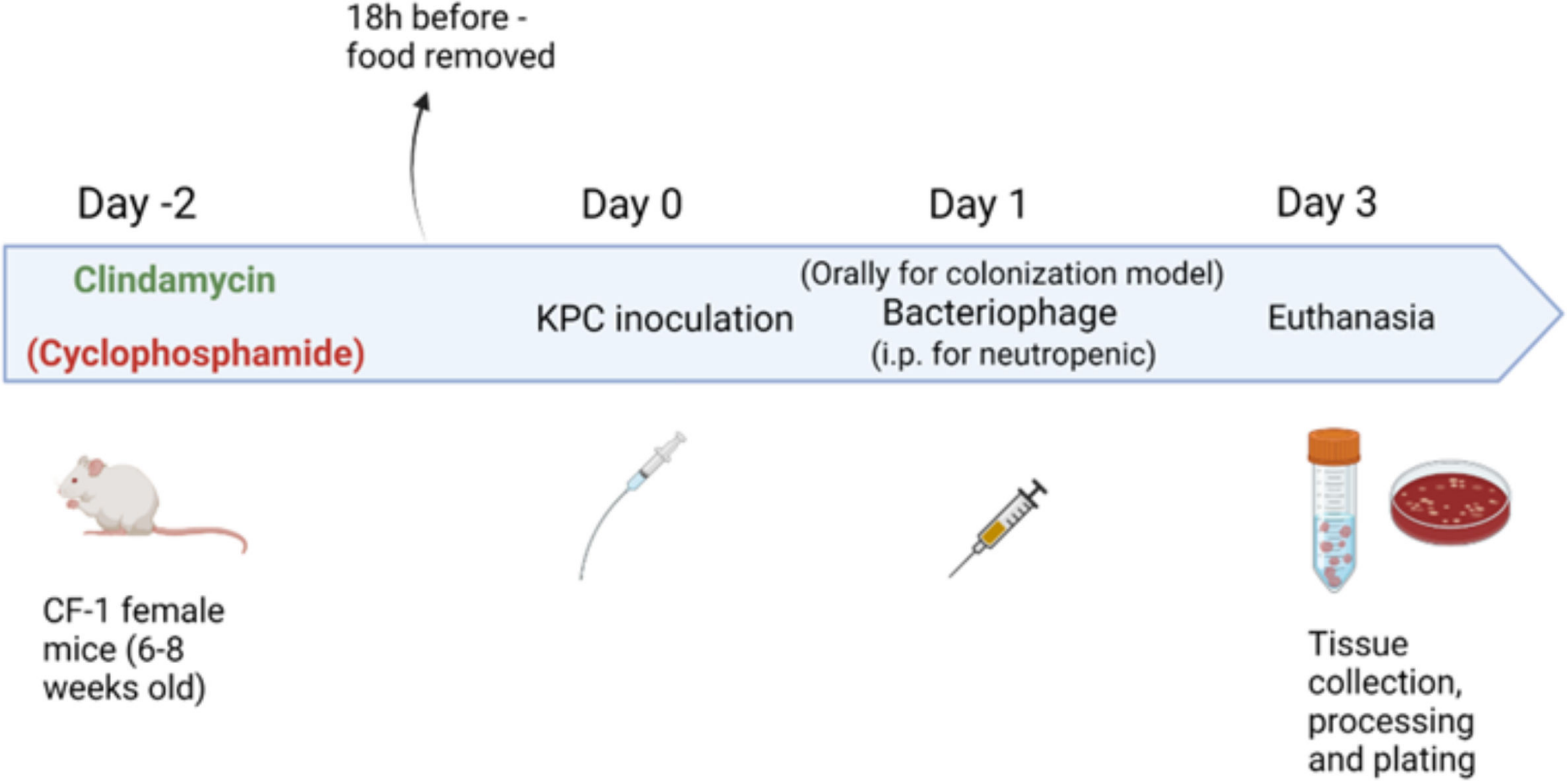
Experimental design for inoculation of neutropenic mice with KPC. Mice were administered IP cyclophosphamide (300 mg/kg) on day −2 and 150 mg/kg on days 0 and 3. On day −2 and on every subsequent day until the end of the experiment, they were administered clindamycin IP (1.5 mg/mouse). On day 0, they were inoculated with KPC via oral gavage and subsequently they received treatment with the three-phage cocktail and/or ceftazidime/avibactam (CAZ/AVI). On day 3, they were euthanized with CO_2_ and their ceca, kidneys and blood were collected for bacterial quantification, cytokine measurement, and phage tittering.

The experimental design for inoculation of neutropenic mice with KPC is described in Fig. 1. The mice were infected with 10^7^ CFU of KPC each via oral gavage, based on a previous protocol established in our laboratory (22). After administering cyclophosphamide at various doses and time points, we concluded that the most consistently neutropenia-inducing regimen was 300 mg/kg intraperitoneally (i.p.) 2 days prior to infection and 150 mg/kg i.p. on days 0 and 3 of the experiment. This cyclophosphamide dosing regimen established the most reproducible neutropenia and did not result in any early deaths of animals due to septicemia. We confirmed that neutropenia was best established with this administration schedule by having no mortality of animals prior to the euthanasia day of the experiment and by always detecting KPC both in the ceca and kidneys of the animals, but not in their blood.

**FIG 2:**
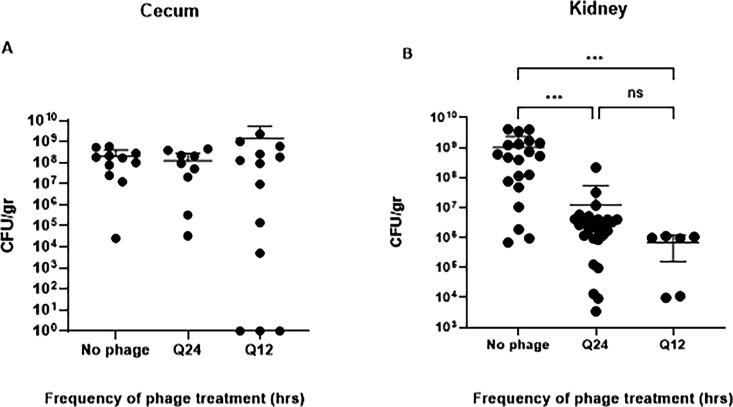
Efficacy of bacteriophage treatment on KPC burden in ceca and kidneys of neutropenic mice. (**A**). Residual bacterial burden (CFU/g) in the cecum of untreated controls and groups treated with 5 × 10^9^ PFU of the three-phage cocktail every 24 h (Q24) and every 12 h (Q12). There were no significant bacterial burden reductions observed with either one of the two bacteriophage treatment regimens in the ceca of mice. (**B**). Residual bacterial burden (CFU/g) in kidney tissue. When treated with 5 × 10^9^ PFU of the three-phage cocktail via i.p. injection every 24 h, a 2 log_10_ CFU reduction was observed (***P = 0.001), whereas a 3 log CFU reduction was observed for animals treated with the same concentration of the three-phage cocktail i.p. every 12 h (***P = 0.001) by Kruskal-Wallis ANOVA. No significant differences were observed in CFU reduction between the Q24 and the Q12 groups.

After reproducible induction of neutropenia, three groups of neutropenic mice infected with KPC were studied: untreated controls, bacteriophage dosed Q24h, and bacteriophage dosed Q12h. Bacteriophage treatment with the phage cocktail, comprised of bacteriophages Pharr, Soft, and Spivey, resulted in an approximately 2 log_10_ reduction in bacterial burden in the kidneys of mice when administered every 24 h (*P* < 0.01), and in an approximately 3 log_10_ reduction when administered every 12 h (*P* < 0.01), in comparison with the untreated controls (Fig. 2). There was no statistically significant difference in CFU reduction between Q24 and Q12. Bacteria that survived phage and were collected from these experiments (*n* = 93) indicated that the majority of bacteria surviving phage treatment remained susceptible to phages Pharr and Soft (Table 1). Resistance to phage Spivey, which is only able to infect Pharr- and Soft-resistant mutants, was not observed.

**TABLE 1:** *In vitro* susceptibility of bacterial colonies recovered following *in viv*o phage treatment to mice^*a*^

Tissue	Dosing interval (h)	Colonies tested	Pharr and Soft susceptible	% Pharr and Soft susceptible	% Spivey susceptible
Kidney	Q12	25	21	84	100
Cecum	Q12	27	19	70	100
Kidney	Q24	21	15	71	100
Cecum	Q24	20	15	75	100
Total		93	70	79	100

^*a*^ After groups of mice received a three-phage cocktail treatment of 5 × 10^9^ PFU every 12 (Q12) and 24 hr (Q24) for 2 days, efficacy in cecum and kidney was determined. A total of 93 KPC colonies were randomly isolated and tested for the potential development of resistance in KPC bacteria against Pharr and Soft. Resistance to phage Spivey, which is only able to infect Pharr- and Soft-resistant mutants, was not observed, as expected due to its design to target only Pharr-Soft-KPC-resistant strains.

### Evaluation of potential prevention of KPC translocation from the gastrointestinal tract to kidneys by the prophylactic administration of bacteriophages

**FIG 3:**
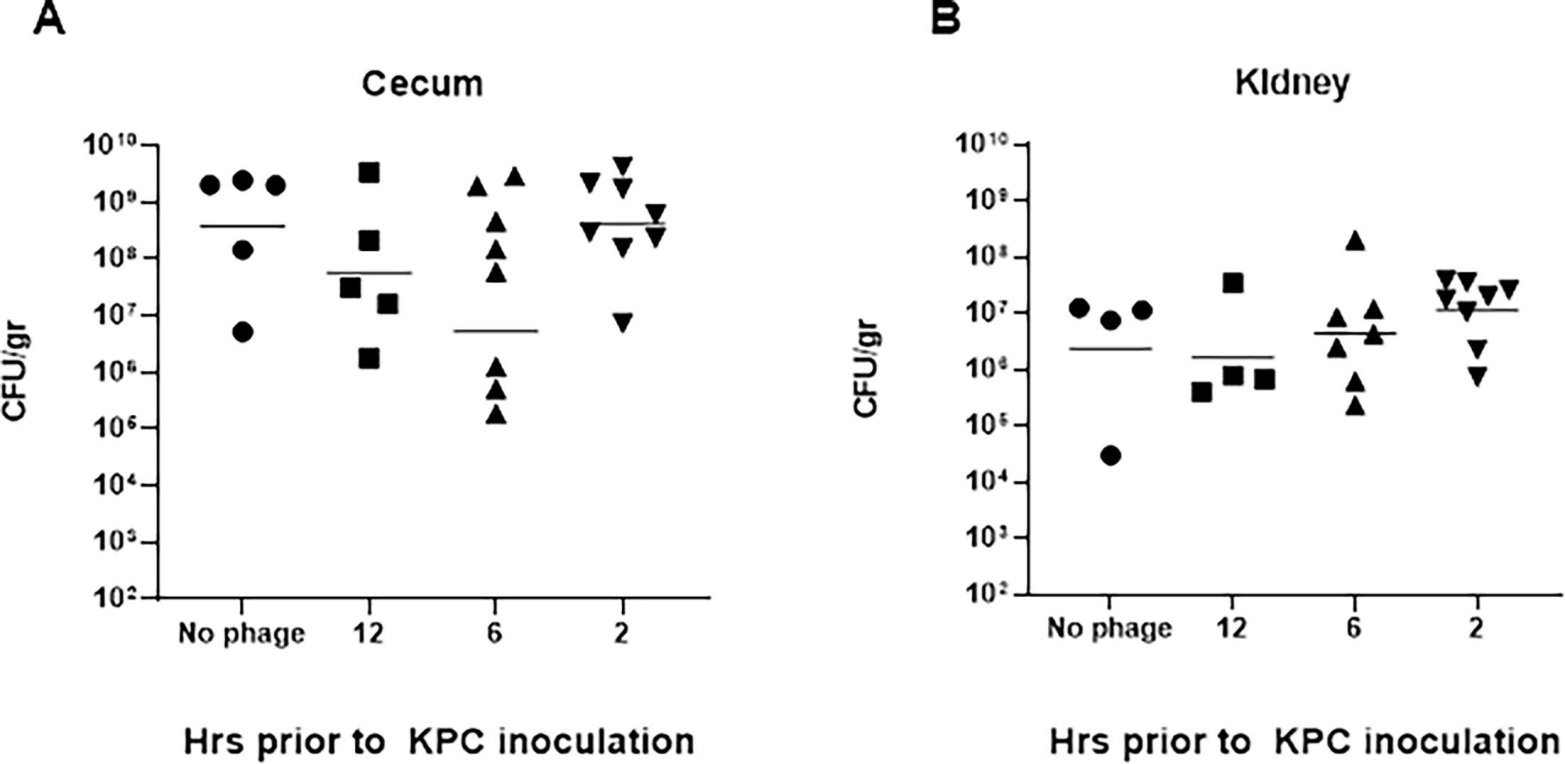
Bacteriophage does not prevent KPC gastrointestinal translocation. (**A**) Cecum CFU/g of pretreated groups with the three-phage cocktail (12 h, 6 h, and 2 h prior to KPC inoculation) and untreated control groups (no phage administration). (**B**) Kidney CFU/g of pretreated groups with phage (12 h, 6 h, and 2 h prior to KPC inoculation) and untreated control groups (no phage administration). Bacteriophage treatment prior to inoculation with KPC did not result in a significant reduction in bacterial burden in cecum and did not prevent translocation to the kidneys.

In order to study the effect of bacteriophages on KPC translocation from the gastrointestinal tract, the three-phage cocktail was administered at different time points (12 h, 8 h, 6 h, and 2 h) to neutropenic mice prior to their inoculation with KPC. These experiments demonstrated that prophylactic administration of bacteriophages did not reduce the KPC burden in the cecum or prevent KPC translocation from the gastrointestinal tract in the neutropenic mouse model (Fig. 3).

### Synergistic interaction between bacteriophages and ceftazidime/avibactam on KPC burden in kidneys of neutropenic mice

**FIG 4:**
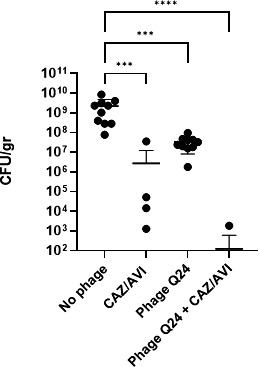
Efficacy of simultaneous administration of the three-phage cocktail and ceftazidime/avibactam on KPC burden in kidneys of neutropenic mice. The combination of bacteriophage (5 × 10^9^ PFU) and CAZ/AVI (80 mg/kg) resulted in synergistic reduction of KPC kidney burden (CFU/gr) in comparison with the untreated control group (****P < 0.001). Treatment with phage only or CAZ/AVI only produced an approximately 2 log_10_ and 2.5 log_10_ reduction, respectively, in KPC kidney burden (***P < 0.01). When all four groups are analyzed by Kruskal-Wallis ANOVA, the relationship is significant (*P* < 0.0001).

Simultaneous administration of the combination of bacteriophages and CAZ/AVI to persistently neutropenic mice infected with KPC resulted in a synergistic reduction of bacterial burden in the kidney (*P* < 0.001) (Fig. 4). Groups 2 and 3, which only received one form of treatment (either CAZ/AVI only or phage only), also showed a lower but significant reduction in bacterial burden (*P* < 0.01).

### Phage–antibiotic interactions *in vitro*

The *in vitro* combination of CAZ/AVI and bacteriophages was neither antagonistic (the antibiotic and the bacteriophages did not negate one another) nor synergistic (Table 2). Phage–antibiotic synergism, which is defined as the resulting reduction in bacterial burden from the combination treatment being larger than the sum of each individual reduction, was observed in our previous study and in other studies (23, 24) but the mechanisms of this interaction remain unclear. Antibiotic sensitivity was found to be largely unchanged between the parental *K. pneumoniae* strain 39427 and its Pharr-resistant (Pharr-R) derivative (Table 3), indicating that the increase in phage resistance is not linked to increased antibiotic sensitivity as has been observed in other systems (25, 26).

**TABLE 2:** Endpoint checkerboard assay incorporating the triple phage cocktail and ceftazidime/avibactam^*a*^

Antibiotic concentration (μg/mL)		Phage concentration (PFU/mL)					
CAZ	AVI	No phage	10^3^	10^4^	10^5^	10^6^	10^7^
16	4	81.4	18.6	17.1	8.6	7.1	8.6
8	2	87.1	12.9	12.9	10.0	12.9	4.29
4	1	88.6	28.6	18.6	14.3	15.7	14.3
2	0.5	97.1	22.9	14.3	14.3	7.14	4.29
1	0.25	102	30.0	14.3	10.0	7.14	8.57
0.5	0.125	101	30.0	15.7	10.0	10.0	7.14
0.25	0.062	100	28.6	17.1	14.3	20.0	7.14
0	0	100	18.6	21.4	18.6	11.4	11.4

^*a*^ The activity of bacteriophage alone (bottom row of the table) markedly reduced the percent growth of KPC *in vitro* by as much as 90% in a concentration-dependent pattern. Similar trends of concentration-dependent bacteriophage activity were observed in the presence of varying concentrations of CAZ/AVI.

**TABLE 3:** Minimum inhibitory concentration assays of the wild-type KPC strain 39427 and its Pharr-resistant derivative 39427 Pharr-R using commercial ESBL broth microdilution assays^*a*^

Antibiotic	Minimum inhibitory concentration	
	39427 wt	39427 Pharr-R
Ceftriaxone	>128	>128
Cephalothin	>16	>16
Cefotaxime	>64	>64
Cefotaxime/clavulanic acid	64/4	64/4
Ceftazidime	>128	>128
Cefazidime/clavulanic acid	>128/4	>128/4
Imipenem	>16	>16
Cefepime	>16	>16
Cefpodoxime	>32	>32
Cefoxitin	>64	>64
Piperacillin/tazobactam	>64/4	>64/4
Meropenem	>8	>8
Gentamicin	4	4
Ciprofloxacin	>2	>2
Ampicillin	>16	>16
Cefazolin	>16	>16

^*a*^ The phage-resistant mutant was not observed to be more susceptible to antibiotics than its parental strain.

### Bacteriophage kinetics in plasma, cecum, and kidney

**FIG 5:**
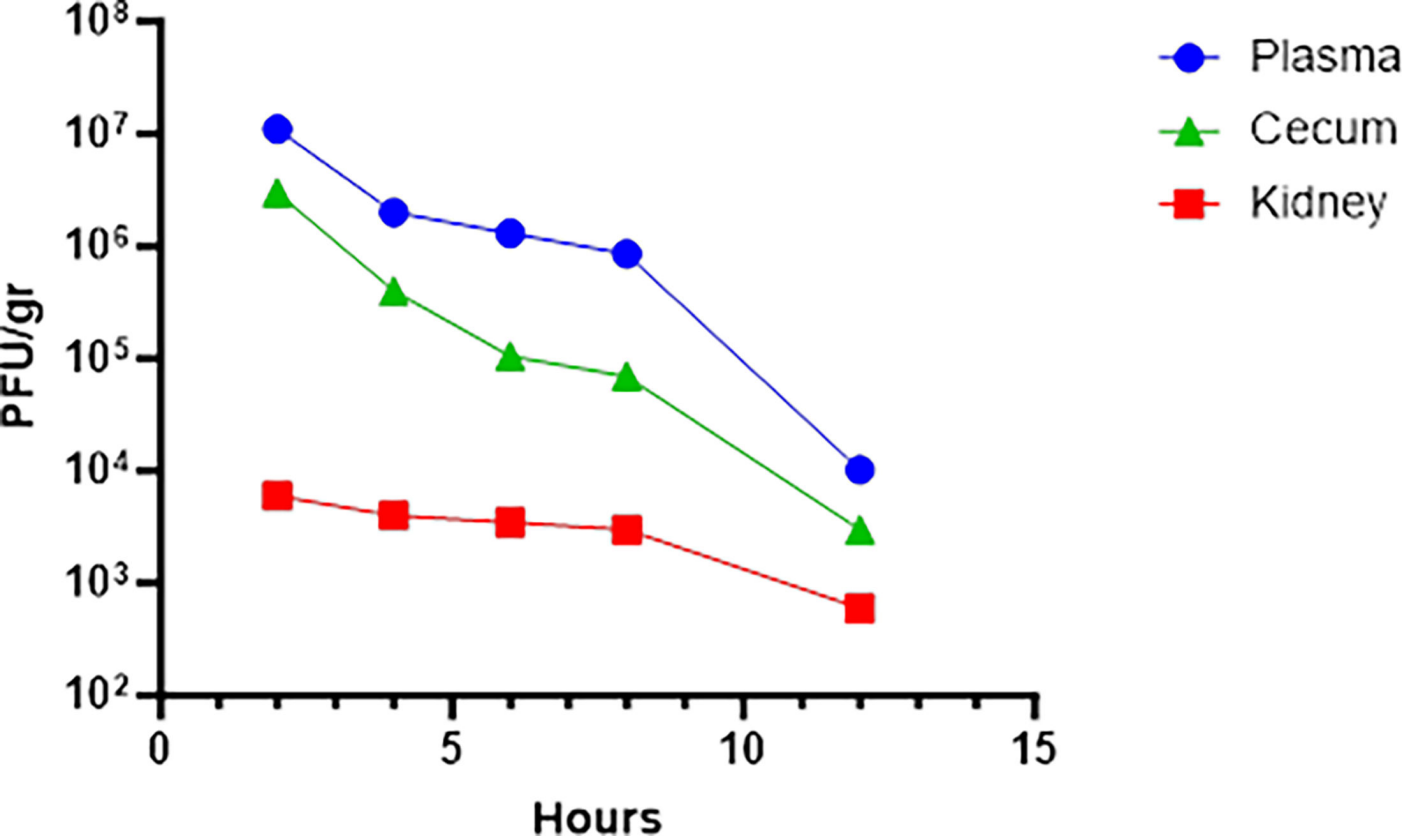
Bacteriophage kinetics in plasma, cecum, and tissue. Mean bacteriophage concentrations of the three bacteriophages in plasma, cecum, and kidney after a single 100 µL i.p. dose of 5 × 10^9^ PFU phage preparation. Titers were reduced linearly on the log scale in plasma, cecum and tissue. The half-life of bacteriophage in the two compartments was 2.5 h.

After collecting plasma, cecum and kidney from animals at 2, 4, 6, 8, 12, and 24 h post-inoculation with KPC, bacteriophage titers were measured with the spot titer method for plasma and kidney, and with the full-plate titering method for cecum. Bacteriophage titers in the plasma were reduced in a linear manner from all three sample types. The two-phase decay calculation of the elimination of bacteriophages with GraphPad Prism software resulted in an elimination rate constant k = 0.0046 and a half-life of 2.5 h (Fig. 5).

### Susceptibility of phage-resistant mutans to complement system killing *in vitro*

**FIG 6:**
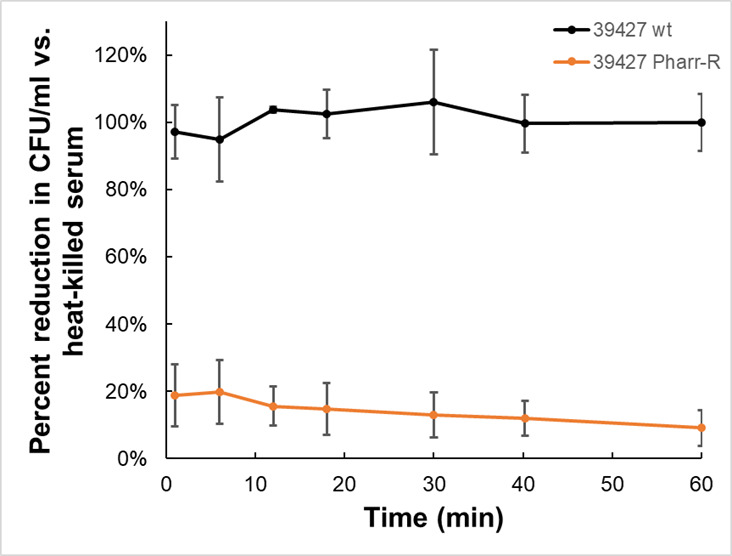
Bacteriophage-resistant KPC mutants are complement-susceptible. KPN39427 WT and the Pharr-resistant 39427 Pharr-R bacterial mutant were treated with either competent human serum or heat-inactivated (HA) human serum and assessed for bacterial survival over a 60 min period. Data shown are adjusted to the number of surviving cells of the same strain incubated in heat-inactivated serum. A mean of three biological replicates is represented, and error bars indicate standard deviation. The wild-type strain 39427 is not affected by complement killing, while the 39427 Pharr-R mutant is sensitive to killing by complement.

**FIG 7:**
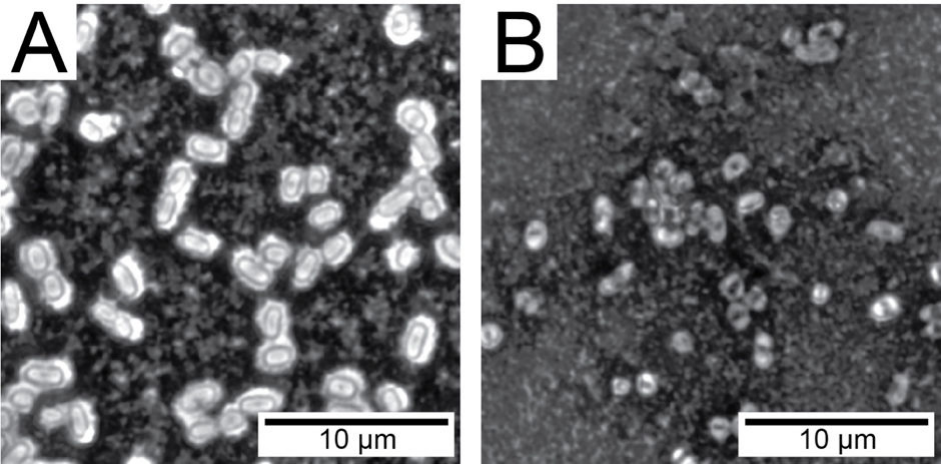
India ink capsule staining of the wild-type *K. pneumoniae* strain 39427 (**A**) and a Pharr-resistant mutant (Pharr-R) (**B**). The presence of capsule is shown in panel A as a clear zone around bacterial cells that excludes the India ink stain. In contrast, the India ink stain is able to travel up to the cell surface in panel B, indicating a lack of capsule.

In order to further understand the effect of phage resistance of KPC on virulence, an *in vitro* serum killing assay was performed to determine the effect of complement on a Pharr-resistant mutant. This mutant demonstrated increased susceptibility to complement killing by human serum (Fig. 6). Resistance to phage Pharr was found by Hesse et al. (27) to be associated with defects in capsule synthesis. The Pharr-resistant mutant used in this study is consistent with these findings, with light microscopy following India ink capsule staining demonstrating the loss of capsule in this phage-resistant mutant (Fig. 7).

### Effects of phage resistance on bacterial fitness *in vivo*

**FIG 8:**
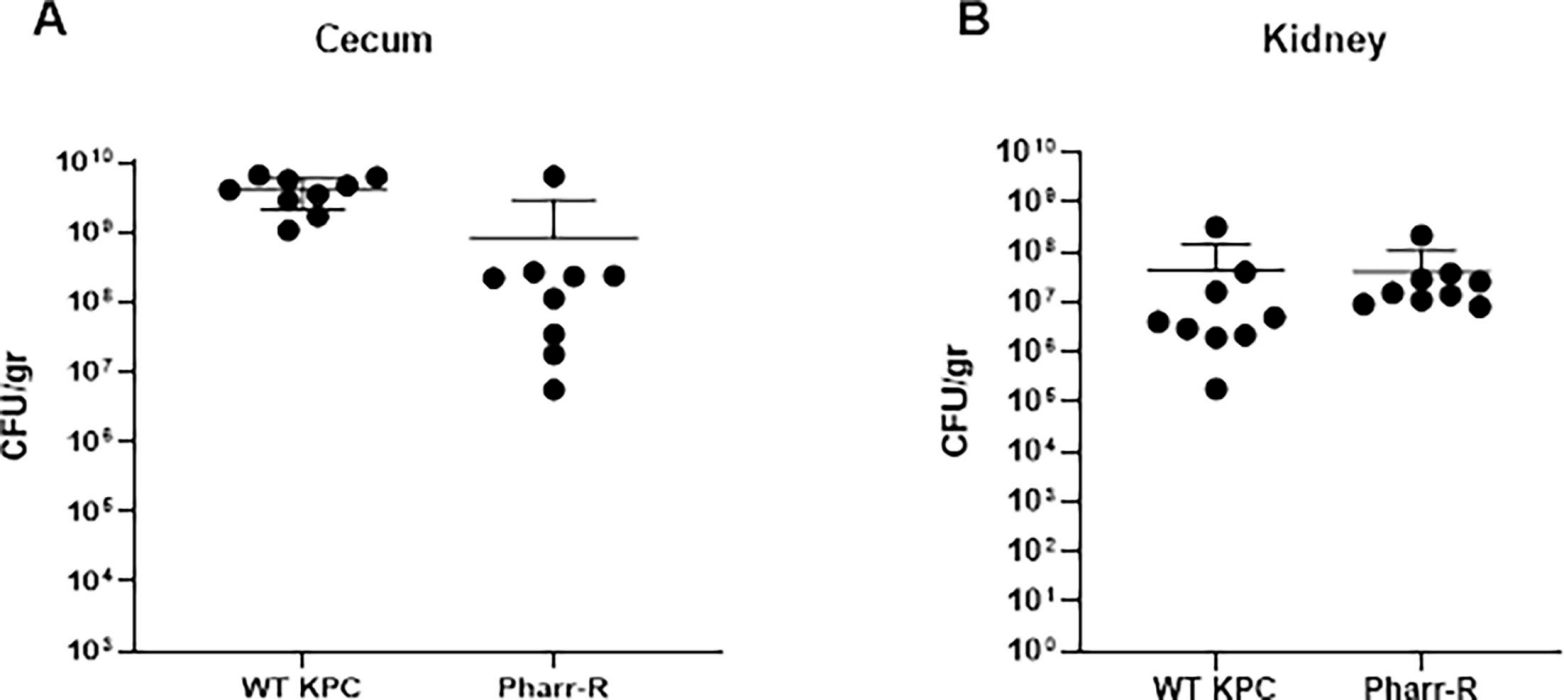
Effects of phage resistance to bacterial virulence *in vivo*. (**A**) Cecum CFU/g, indicative of the colonization ability (and therefore the virulence) of 39427 Pharr-R) derived from the phage-treated groups, in comparison with the WT KPC. (**B**) Kidney CFU/g, indicative of the extent of translocation of the Pharr-R derived from phage-treated mice, in comparison with the WT KPC.

Structural changes in some strains of KPC that are resistant to bacteriophages may result in a loss of fitness. We therefore examined whether the loss of capsule observed in the Pharr-resistant mutant of KPC affected the ability of the bacteria to colonize mouse tissues. Neutropenic mice, premedicated with clindamycin and cyclophosphamide following the protocol for KPC translocation, were inoculated via oral gavage with either 10^7^ CFU of the wild-type KPC or the Pharr-R. The ability of Pharr-R of KPC to colonize the cecum, translocate and infect the kidney, was not significantly affected by the loss of capsule. This mutant displayed residual CFUs within the renal tissue that were comparable to those of the wild-type KPC (Fig. 8).

### Cytokine measurement and investigation of the immune response of the mice to KPC infection and/or bacteriophage administration

To study the immune response of the mice and evaluate the effects of bacterial infection or phage-only administration on the immune system, we measured the pro-inflammatory cytokines in plasma after administration of the three-phage cocktail only or the three-phage cocktail and CAZ/AVI. We also investigated the plasma cytokine levels after administration of the Pharr-R mutant to assess potential differences due to the loss of capsule and the potential exposure of the other structures of the outer membrane to the immune cells of the mice.

**FIG 9:**
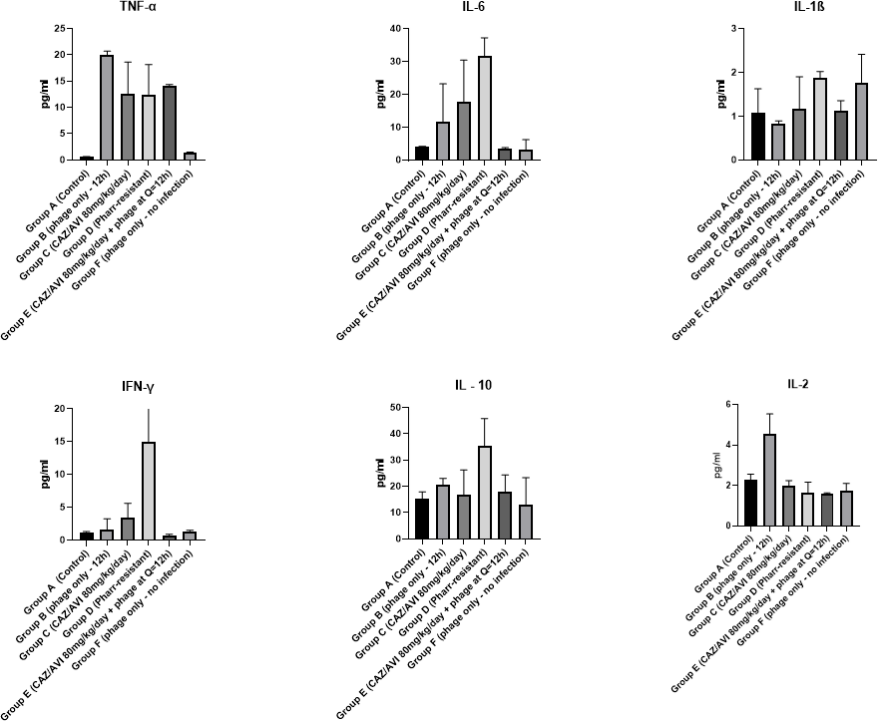
Cytokine measurement and investigation of the immune response of the mice to KPC infection and/or bacteriophage administration. Cytokines were measured in plasma of mice, using the MSD V-plex pro-inflammatory mouse panel, after blood was collected from the animals in EDTA tubes with cardiac puncture. Tumor necrosis factor-alpha (TNF-α) was elevated in all groups that were infected with KPC, whether it was the WT or the Pharr-R mutant, regardless of whether they were treated with bacteriophage and/or CAZ/AVI. The phage-only group, which received only the three-phage cocktail (Pharr, Soft, and Spivey), did not exhibit increased TNF-α levels. None of the groups had increased levels of interferon-γ (IFN-γ), except for the group which was inoculated with the Pharr-R mutant of KPC. No definitive conclusions can be drawn for inteleukin-1 β (IL-1β) or interleukin-2 (IL-2) as all groups had similar levels of these cytokine. IL-6 was significantly increased in the Pharr-R-infected group, which received no treatment, whereas it was decreased compared to that group, in all treated groups, among which treatment with both bacteriophage and CAZ/AVI seemed to have the most impact on the reduction of this cytokine. Treatment with bacteriophage only, CAZ/AVI only, or a combination of both, slightly reduced IL-10, compared with the Pharr-R-challenged group, which received no treatment.

In general, a phage-only administration with no bacterial infection did not induce significant cytokine production in relation to other groups, indicating that these phages are either not detected by immune cells or they are taken up by macrophages or dendritic cells with minimal inflammatory reaction (Fig. 9).

By comparison, treatment of KPC infection with bacteriophage, CAZ/AVI, or the combination resulted in increased plasma levels of TNF-α and IL-6 in comparison with those untreated infected controls and phage-only-treated mice (Fig. 8). There was no notable change in the plasma levels of IFN-γ or IL-10 in these treated groups. There was considerable intergroup variability in expression of IL1-β.

Finally, administration of Pharr-R induced the excretion of TNF-α, IL-6, or IFN-γ two- to fivefold compared with the control group, suggesting exposure of LPS and pro-inflammatory cell wall components (Fig. 9).

## DISCUSSION

This study demonstrates the efficacy of a bacteriophage cocktail comprised of three phages against KPC renal infection in a neutropenic murine model of gastrointestinal translocation and further demonstrates synergistic interaction with CAZ/AVI. This phage cocktail reduced KPC burden in kidneys by approximately 2 log_10_ (*P* < 0.01) when administered every 24 h. This effect was pharmacodynamically enhanced by more frequent administration of the phage cocktail (every 12 h), resulting in a 3 log_10_ reduction of renal KPC burden (*P* < 0.01). Despite its marked effectiveness against renal infection, prophylactic administration of the phage cocktail was not shown to be efficient in preventing KPC translocation in neutropenic murine hosts. Notably, even among mice with a lower range of residual bacterial burden in cecum (approximately 10^5^ CFU/g), there was sufficient KPC to result in renal translocation in neutropenic hosts.

Previous studies of bacteriophages for the treatment of KPC infection have demonstrated *in vivo* activity in immunocompetent acutely septic murine models infected through an intraperitoneal route (16, 18). The studies reported herein provide new information on the efficacy of phage therapy in immunocompromised animals. Our neutropenic murine model of KPC infection recapitulates the infection in immunocompromised patients receiving intensive cytoreductive chemotherapy. The model more accurately reflects the pathogenesis of KPC infection in patients with hematological malignancies with bacterial translocation across the gastrointestinal tract and dissemination to deep tissue infection. Moreover, our model is a subacute nonlethal infection that extends to 3 days of survival, allowing more time to understand the therapeutic effect as may be observed with the treatment of clinical infections. Our study also characterizes the plasma bacteriophage pharmacokinetics and cytokine response to infection and treatment. Finally, we demonstrate synergistic interaction between bacteriophages and an active antimicrobial, which has not been previously demonstrated *in vivo* against disseminated infection caused by KPC.

Bacteriophage treatment when applied alone demonstrated a significant reduction in bacterial burden in kidneys. When used in combination with CAZ/AVI, bacteriophage treatment produced a synergistic eradication of renal infection. This efficacy of bacteriophage treatment establishes a preclinical foundation for further investigation of this modality in immunocompromised patients with serious KPC infections.

The burden of infection in this subacute model of KPC bacterial translocation results in the preponderance of infection in the kidneys. No organisms were detected in the bloodstream above the lower limit of detection in our quantitative cultures. Although an acute septic model of intravenously administered KPC would disseminate organisms in relatively large concentrations into other tissue sites, that model would not be relevant to our study due to its high mortality. The model of KPC gastrointestinal translocation reflects both a common portal of infection in immunocompromised patients and the ability to assess the impact of bacteriophage on gastrointestinal decolonization and translocation.

As most patients with KPC infection will be receiving an antimicrobial agent (8), understanding the potential for antagonistic or synergistic interaction between CAZ/AVI and phage is important. The *in vivo* data presented herein illustrated a significant reduction in renal KPC burden (nearly 5 log_10_) following treatment by a combination of phage and antibiotic. There are several possible mechanisms for this synergistic activity. Ceftazidime inhibits peptidoglycan synthesis in the cell call by binding to penicillin-binding proteins, while bacteriophages produce lysins that directly hydrolyze the peptidoglycan bonds. However, as the *in vivo* synergistic effect between CAZ/AVI and bacteriophage was not apparent in the *in vitro* checkerboard assay, there are likely other factors, such as complement, mannose-binding lectin, or other soluble or tissue-based mediators that contribute to this potent *in vivo* interaction.

The combination of these bacteriophages and CAZ/AVI warrants further investigation as a potentially powerful combination in the treatment of patients with serious KPC infections. Previous work in our laboratory also demonstrated a synergistic reduction by the three-phage cocktail plus CAZ/AVI of KPC colonization in the murine gastrointestinal tract (22). The recent emergence of resistance of KPC strains to CAZ/AVI further underscores the therapeutic potential of bacteriophages against these pathogens (28, 29).

Bacteria are in a constant evolving race to face the challenges posed to them by their natural predators. As a result, bacteriophage resistance can potentially occur during the course of bacteriophage treatment (30). A common hypothesis governing past years of bacteriophage research proposed that phage-resistant mutants that would result from treatment would display increased susceptibility to antibiotics (27, 31). The mechanism for this increased susceptibility to antibacterial agents is attributed to alterations in their outer cell membrane and/or capsule, which would permit higher concentrations of antibiotics to enter the bacterial cell and to exert their activity (32). Defects in capsule synthesis are known to confer resistance to phage Pharr (27, 31) and by extension to phage Soft, which is unable to infect Pharr-resistant mutants. However, the majority of the tested bacterial clones collected from phage-treated animals showed that most were still susceptible to Pharr and Soft (Table 1), suggesting that bacterial avoidance of phage, such as in protected sites, may be more of a limiting factor in treatment than was the emergence of resistance. When studying potential MIC changes of several antimicrobial agents against the phage-resistant mutant, no changes in susceptibility were observed, indicating that the occurrence of bacteriophage resistance does not necessarily result in increased susceptibility to conventional antibiotics.

When further exploring the potential mechanism behind the observed *in vivo* synergy in an *in vitro* experiment of complement system killing in serum, it was observed that the phage-resistant mutant became more susceptible to complement. Since the pathogen that we used in all experiments is a human pathogen and has been isolated from human patients, we investigated its susceptibility to the complement system in human serum. Isolates of *K. pneumoniae* are variably susceptible to complement killing (33, 34). Opstrup and colleagues (34) and Jensen et al. (35) demonstrated that serum-sensitive isolates may be killed by the classical/lectin pathways, as well as by the alternate pathway. Some serum-resistant isolates of *K. pneumoniae* were found to bind the components of complement to the capsules. This observation highlights a potential mechanism for loss of fitness *in vivo*, which would include reduced protection from complement killing due to the loss of capsule (36, 37).

Our studies of bacteriophage kinetics in plasma, cecum, and kidney tissues of infected mice revealed a linear reduction on the logarithmic scale, with a half-life of 2.5 h in plasma and cecum and kidneys and sufficient bacteriophage titers in renal tissue to potentially exert sustained therapeutic effect (Fig. 5).

In summary, this work highlights the efficacy of bacteriophage treatment, alone or in combination with antibiotics, against renal KPC infection in a neutropenic murine model and has important clinical implications for the treatment of these lethal infections in neutropenic patients with the potential to be used alone or in combination with existing small molecule antimicrobial agents.

## MATERIALS AND METHODS

### Bacterial strains

The ST258 *K. pneumoniae* bloodstream clinical isolate 39427 (GenBank NZ_NDBM01000000 and NZ_CP054268) was used for all murine challenge studies (3). The KPC strain 39427 (3) was kindly provided by Dr. Michael Satlin (Weill Cornell Medicine) and previously studied in our laboratory (22, 38, 39). This strain and its phage Pharr-resistant derivative 39427 Pharr-R were used for all *in vitro* and *in vivo* experiments in the murine model. *K. pneumoniae* strain 1776pc is an ST258 strain cured of its KPC plasmid and kindly provided by Dr. Karen Frank (NIH Clinical Center), and this strain and its Pharr-resistant derivative were used for large-scale culture of phages for use in *in vivo* experiments. Bacteria were routinely cultured on tryptic soy broth (TSB, Difco) or tryptic soy agar (TSA, Difco) aerobically at 37°C. The MIC of CAZ/AVI for strain 39427 is 2 µg/mL (38, 39).

### Preparation of bacterial inoculum

In preparation of the KPC inoculum, bacteria were prepared by suspending a bacterial colony from TSA agar into 50 mL of TSB and shaking at 37°C for 4 h. When an optical density of 0.1 at 600 nm (OD_600_ = 0.1) was reached, corresponding to approximately 10^8^ CFU/mL, the culture was centrifuged at 5,000 × *g* for 5 min, the supernatant was discarded, and the resulting pellet was resuspended twice in sterile normal saline (0.9% NaCl). OD_600_ was measured again to 0.1, and 100 µL of the resulting inoculum (equaling 10^7^ CFU) were administered to the animals via oral gavage.

### Bacteriophages

In this study, we investigated the efficacy of bacteriophage treatment in a neutropenic murine model of KPC gastrointestinal translocation on the bacterial burden in renal infection. We utilized three diverse virulent bacteriophages: Pharr (family *Autographiviridae*), Soft (family *Casjensviridae*), and Spivey (family *Demerecviridae*). Pharr, Soft, and Spivey were administered to infected neutropenic mice in combination in order to minimize emergence of KPC resistance to individual bacteriophages. Pharr and Soft were initially identified from among a group of nine phages that infected the KPC + ST258 *K*. *pneumoniae* strain 39427, and phage Spivey was isolated against a phage Pharr-resistant derivative of strain 39427 (22, 40). Sequence analysis demonstrated that these isolates were strictly lytic phages and contained no bacterial virulence factors, toxins, or integrases.

Bacteriophages Pharr (NC_048175), Soft (NC_048805), and Spivey (MK630230) were used in all experiments. Phages were routinely titered by the soft agar overlay method (41) using TSA bottom plates and top agar consisting of 10 g/L Bacto tryptone (VWR), 10 g/L NaCl, and 0.5% Bacto agar (VWR), supplemented with 5 mM each of CaCl_2_ and MgSO_4_. Host lawns were established by inoculating 4 mL of molten top agar with 0.1 mL of a mid-log (OD_550_ 0.3–0.5) KPC host culture. For administration in murine models, phage lysates were prepared by inoculating log-phase, 1 L TSB liquid cultures of *K. pneumoniae* strain 1776pc (for phages Pharr and Soft) or 1776pc Pharr-R (for phage Spivey) with phage at a multiplicity of infection of 0.001–0.1 and culturing until lysis. Phage lysates were cleared by centrifugation (8,000 × *g*, 4°C, 10 min), filter-sterilized, and purified by isopycnic CsCl gradient ultracentrifugation as described previously (42). Residual endotoxin was removed by passage through an Endotrap HD column (Lionex), filter-sterilized, and final endotoxin was determined to be less than 1 EU/mL by EndoZyme II recombinant factor C assay (bioMerieux). A combination (cocktail) of the three phages at a total concentration of 5 × 10^10^ PFU was used in all experiments.

### Animals

Female 6- to 8-week-old CF-1 mice weighing 26–30 g (Charles River Laboratories) were used in all experiments. Mice at that age are considered adults, and their weight allowed for reproducible data and easier dosing calculations. Outbred mice were used, aiming for an extrapolation of the data to the genetic diversity that is observed in humans.

### KPC colonization and translocation in neutropenic mice

Female, 6- to 8-week-old CF-1 mice, weighing 26–30 g, were rendered neutropenic by administration of cyclophosphamide, and resident gastrointestinal bacterial microbiome was disrupted by administration of clindamycin prior to inoculation with KPC strain 39427 via oral gavage. The day of KPC inoculation was designated as day 0 of the experiment. Mice were administered 300 mg/kg cyclophosphamide IP on day −2, and 150 mg/kg cyclophosphamide IP on each of days 0 and 3 (Fig. 1). Starting on day −2, mice also received a loading dose of clindamycin 1.5 mg IP per mouse. Each subsequent day until the end of the experiment, they received clindamycin 1.5 mg/kg IP. Eighteen hours prior to KPC inoculation, food was removed from mouse enclosures to prevent coprophagy. On day 0, a KPC inoculum of 10^7^ CFU/mouse (0.1 mL of an OD = 0.1 culture, corresponding to 10^8^ CFU/mL) was administered to the mice via oral gavage, using a blunt 20-gauge oral gavage needle. On days 1 and 2 of the experiment, infection was confirmed by plating stool of the mice on HardyCHROM CRE agar plates (Hardy Diagnostics). Animals were monitored twice daily for humane endpoints, including hunched posture, squinting of the eyes, lethargy, ruffled fur, and weight loss. Whenever an animal exhibited such endpoints, it was euthanized and cecum, kidneys, and plasma were collected for bacterial quantification and inflammatory biomarker measurement. Animals only exhibited such symptoms in the initial phases of the experiment, when standardizing inocula. During the treatment phases of the experiments, no animals died prior to the final day of the experiment. Therefore, the data do not depict tissue bacterial burdens of animals that died prematurely. On day 3 of the experiment, animals were euthanized by CO_2_ overdose, and their cecum and kidneys were collected in sterile normal saline (0.9% NaCl). Blood was collected via cardiac puncture. The ceca and kidneys of the mice were weighed and homogenized using a tissue homogenizer (Omni). Tissue homogenates were serially diluted and plated on CRE selective agar plates (HardyCHROM) using the single-plate serial dilution spotting (SP-SDS) method (43). Plates were incubated at 37°C overnight, colonies with the expected dark blue CRE morphology were enumerated, and counts adjusted to CFU/g sample. The lower limit of detection of the assay was 10^2^ CFU/g.

### Prophylactic model of KPC translocation

In an effort to explore the possibility of prevention of KPC translocation with oral administration of bacteriophages, 5 × 10^9^ PFU of bacteriophage was administered via oral gavage to the mice, at three different time points: 12, 6, and 2 h prior to inoculation with KPC. The mice were euthanized 24 h after KPC inoculation, ceca and kidneys were collected as described previously, and CFU were determined as mentioned.

### Simultaneous administration of CAZ/AVI and bacteriophage on bacterial burden in cecum and kidney

Animals were assigned to four study groups: the first group received no antibacterial treatment (untreated controls), the second group received CAZ/AVI (80 mg/kg/day IP divided in three doses), the third group received a combination of CAZ/AVI (80 mg/kg/day IP) and bacteriophage (5 × 10^9^ PFU/mouse), and the fourth group received bacteriophage-only treatment (5 × 10^9^ PFU). The experiment was conducted for 4 days, on which time the mice were euthanized with CO_2_, their ceca and kidneys collected, and CFU determined as previously described.

### Virulence studies

The effect of structural changes on the virulence of phage-resistant KPC mutants was examined by inoculating different groups of mice with each mutant and then performing quantitative cultures of KPC in kidneys and ceca. Group 1 was a wild-type-infected group, and group 2 was inoculated with the Pharr-R mutant.

### Plasma sample preparation

Blood samples collected in EDTA tubes were centrifuged at 4°C, 4,000 rpm for 15 min. Plasma was collected with a pipette, transferred to screw top 1.5 mL tubes (VWR) and stored at −80°C.

### Serum bactericidal assay

KPC bacterial cultures diluted to 10^5^ CFU/mL in TSB and placed on ice were incubated in both serum and heat-inactivated serum (Sigma-Aldrich) at 1:10 ratio for final concentration estimated at 10^4^ CFU/mL. Cells were incubated at 37°C with time points taken and plated on TSA plates for surviving CFU. The lower limit of detection was 50 CFU/mL.

### Bacteriophage susceptibility of phage-resistant KPC isolates recovered following *in vivo* treatment

Colonies cultured from murine kidney or cecal samples following multiple doses (Q24, Q12) of bacteriophage in the clindamycin and cyclophosphamide-immunosuppressed murine model of KPC infection were cultured and susceptibility to bacteriophages Pharr, Soft and Spivey was conducted by spot titer assay.

### Antimicrobial susceptibility of WT and phage-resistant mutants

Bacterial susceptibility to antimicrobial agents was determined by microplate MIC assays using standard ESBL assay plates (Trek Diagnostics). Bacterial inoculum was standardized to between 1 × 10^5^ and 1 × 10^6^ CFU using a 0.5 McFarland turbidity standard and immediate serial dilution, with inoculated CFU/mL confirmed by plating to TSA plates. To each well, 100 µL of inoculum was added to each well of the ESBL plate, the plate was sealed, and incubated at 37°C for 18 h before being read. Each well was given either a positive or negative score for growth, defined as the well having any growth being positive. From these positive/negative scores, the actual MIC was generated with the lowest concentration of antibiotic that inhibited bacterial growth being considered the MIC for that strain/antibiotic combination. The data are representative of two technical replicates.

### Checkerboard assay for *in vitro* evaluation of the efficacy of phage and CAZ/AVI combination

A checkerboard assay representing combinations of bacteriophage and CAZ/AVI was applied to determine growth inhibition of single agents and combinations following an 18 h incubation. Combinations included the bacteriophage at 10^7^ to 10^3^ PFU or No Phage, and CAZ/AVI from 16 to 0.025 µg/mL or no antibiotic. Bacterial cultures were added at 5 × 10^5^ CFU final concentration as described previously. Final well volumes were 200 µL and plates were covered and incubated 18 h at 37°C before results were read using a plate reader measuring absorbance at 550 nm. Bacterial strains tested were 39427 WT and 39427 Pharr-R. Results shown represent three averaged replicates.

### Bacteriophage kinetics in plasma, cecum, and kidneys

After euthanasia of mice, their blood was collected via cardiac puncture and plasma was isolated as described above. Their cecum and kidneys were also collected and homogenized using an Omni homogenizer. Cecal and renal tissue homogenates were treated by mixing in the presence of 5% (v/v) CHCl_3_, centrifuging to pellet the solid material, and plating the serially diluted the supernatants for phage by spot titer on soft agar overlays. Elimination of bacteriophages from plasma, cecum, and kidneys was determined by t1/2 = 0.693/k, where k = the elimination rate constant and t1/2 = halflife by (GraphPad Prism, Boston, MA).

### Cytokine measurement and investigation of the immune response of the mice

Blood was collected from mice right after euthanasia with CO_2_, via cardiac puncture. After CO_2_ euthanasia, the animal was placed on a solid surface with its ventrum exposed. The xyphoid process was palpated at the caudal aspect of the sternum and a notch was palpable on both sides of this process. A 1¨ 22 gauge or larger needle attached to a 1–3 mL syringe is inserted into either notch and directed toward the midline at an approximately 40° angle. Negative pressure was applied by pulling gently back on the plunger after it had been inserted under the skin. Once blood collection is completed, death is verified by confirming the absence of a heartbeat by palpation by placing the thumb and index finger on opposing sides of the animal’s chest. If a heartbeat is detected, euthanasia will be completed by exposure to carbon dioxide for at least 15 min.

After collecting the blood in EDTA tubes to prevent clotting, the tube was gently inverted several times to mix the blood with the anticoagulant. The tubes were centrifuged at 1,200–1,500 × *g* for 10–15 min at room temperature. After centrifugation, the blood was separated into three layers: plasma (top layer), buffy coat (middle layer containing white blood cells), and red blood cells (bottom layer). Plasma was carefully pipetted from the top layer without disturbing the buffy coat or red blood cells.

Plasma samples and all reagents from the MSD V-plex pro-inflammatory mouse panel were taken out of the freezer and left to return to room temperature. Calibrators, controls, and samples were prepared according to the protocol provided by the company. The 96-well plate, which was pre-treated with the capture antibodies, was washed three times and 50 µL of the calibrators, samples and controls were added in each well. The plate was washed again three times, and the detection antibodies were added. After sealing the plate with adhesive tape, we left it at room temperature under shaking for a 2 h incubation. Following that, the plate was washed again three times, and read buffer was added to the wells, after which the plate was read by the MSD 1300 Microplate Reader SQ120. The MSD software generated a report with the results, which were then plotted into graphs per different cytokine.

Cytokines were measured for the following animal groups: group A (control) was a non-KPC-infected group, which additionally did not receive either bacteriophage or CAZ/AVI treatment. Group B was a KPC-infected group, which received treatment with the three-phage cocktail (Pharr, Soft, and Spivey) every 12 h (Q = 12 h), via the i.p. route. Group C was a KPC-infected group, which received i.p. treatment with CAZ/AVI at a dose of 80 mg/kg/day. Group D was a KPC-infected group, which did not receive either bacteriophage or CAZ/AVI treatment. Group E was a KPC-infected group, which received combination treatment with both the three-phage cocktail (Pharr, Soft, and Spivey) (5 × 10^9^ PFU/animal. Q = 12 h) and CAZ/AVI (80 mg/kg/day), and group F was a non-KPC-infected group, which only received bacteriophage administration of the three-phage cocktail.

### Statistical analysis

Data were analyzed by non-parametric measures of continuous variables. Mann-Whitney U test was used for comparisons of continuous variables between two groups and Kruskal-Wallis test was used to compare continuous variables among all groups. A two-tailed *P* value of < 0.05 was considered to be statistically significant.

## References

[1] Chen L, Mathema B, Chavda KD, DeLeo FR, Bonomo RA, Kreiswirth BN. 2014. Carbapenemase-producing Klebsiella pneumoniae: molecular and genetic decoding. Trends Microbiol 22:686–696. doi:10.1016/j.tim.2014.09.003 25304194 PMC4365952

[2] Munoz-Price LS, Poirel L, Bonomo RA, Schwaber MJ, Daikos GL, Cormican M, Cornaglia G, Garau J, Gniadkowski M, Hayden MK, Kumarasamy K, Livermore DM, Maya JJ, Nordmann P, Patel JB, Paterson DL, Pitout J, Villegas MV, Wang H, Woodford N, Quinn JP. 2013. Clinical epidemiology of the global expansion of Klebsiella pneumoniae carbapenemases. Lancet Infect Dis 13:785–796. doi:10.1016/S1473-3099(13)70190-7 23969216 PMC4673667

[3] Satlin MJ, Chen L, Patel G, Gomez-Simmonds A, Weston G, Kim AC, Seo SK, Rosenthal ME, Sperber SJ, Jenkins SG, Hamula CL, Uhlemann A-C, Levi MH, Fries BC, Tang Y-W, Juretschko S, Rojtman AD, Hong T, Mathema B, Jacobs MR, Walsh TJ, Bonomo RA, Kreiswirth BN. 2017. Multicenter clinical and molecular epidemiological analysis of bacteremia due to carbapenem-resistant Enterobacteriaceae (CRE) in the CRE epicenter of the United States. Antimicrob Agents Chemother 61:e02349-16. doi:10.1128/AAC.02349-16 28167547 PMC5365653

[4] Bowers JR, Kitchel B, Driebe EM, MacCannell DR, Roe C, Lemmer D, de Man T, Rasheed JK, Engelthaler DM, Keim P, Limbago BM. 2015. Genomic analysis of the emergence and rapid global dissemination of the clonal group 258 Klebsiella pneumoniae pandemic. PLoS One 10:e0133727. doi:10.1371/journal.pone.0133727 26196384 PMC4510304

[5] Rojas LJ, Salim M, Cober E, Richter SS, Perez F, Salata RA, Kalayjian RC, Watkins RR, Marshall S, Rudin SD, Domitrovic TN, Hujer AM, Hujer KM, Doi Y, Kaye KS, Evans S, Fowler VG Jr, Bonomo RA, van Duin D, Antibacterial Resistance Leadership Group. 2017. Colistin resistance in carbapenem-resistant Klebsiella pneumoniae: laboratory detection and impact on mortality. Clin Infect Dis 64:711–718. doi:10.1093/cid/ciw805 27940944 PMC5850634

[6] Cuzon G, Naas T, Truong H, Villegas MV, Wisell KT, Carmeli Y, Gales AC, Venezia SN, Quinn JP, Nordmann P. 2010. Worldwide diversity of Klebsiella pneumoniae that produce β-lactamase bla_KPC-2_ gene. Emerg Infect Dis 16:1349–1356. doi:10.3201/eid1609.091389 20735917 PMC3294963

[7] Zhang X, Li F, Cui S, Mao L, Li X, Awan F, Lv W, Zeng Z. 2020. Prevalence and distribution characteristics of bla_KPC-2_ and bla_NDM-1_ genes in Klebsiella pneumoniae. Infect Drug Resist 13:2901–2910. doi:10.2147/IDR.S253631 32903853 PMC7445519

[8] Bassetti M, Peghin M. 2020. How to manage KPC infections. Ther Adv Infect Dis 7:2049936120912049. doi:10.1177/2049936120912049 32489663 PMC7238785

[9] Hobson CA, Pierrat G, Tenaillon O, Bonacorsi S, Bercot B, Jaouen E, Jacquier H, Birgy A. 2022. Klebsiella pneumoniae carbapenemase variants resistant to ceftazidime-avibactam: an evolutionary overview. Antimicrob Agents Chemother 66:e0044722. doi:10.1128/aac.00447-22 35980232 PMC9487638

[10] Montrucchio G, Corcione S, Sales G, Curtoni A, De Rosa FG, Brazzi L. 2020. Carbapenem-resistant Klebsiella pneumoniae in ICU-admitted COVID-19 patients: keep an eye on the ball. J Glob Antimicrob Resist 23:398–400. doi:10.1016/j.jgar.2020.11.004 33242674 PMC7682477

[11] Satlin MJ, Jenkins SG, Walsh TJ. 2014. The global challenge of carbapenem-resistant Enterobacteriaceae in transplant recipients and patients with hematologic malignancies. Clin Infect Dis 58:1274–1283. doi:10.1093/cid/ciu052 24463280 PMC4038783

[12] Qin X, Wu S, Hao M, Zhu J, Ding B, Yang Y, Xu X, Wang M, Yang F, Hu F. 2020. The colonization of carbapenem-resistant Klebsiella pneumoniae: epidemiology, resistance mechanisms, and risk factors in patients admitted to intensive care units in China. J Infect Dis 221:S206–S214. doi:10.1093/infdis/jiz622 32176790

[13] Chen LF, Anderson DJ, Paterson DL. 2012. Overview of the epidemiology and the threat of Klebsiella pneumoniae carbapenemases (KPC) resistance. Infect Drug Resist 5:133–141. doi:10.2147/IDR.S26613 23055754 PMC3460674

[14] Tesfa T, Mitiku H, Edae M, Assefa N. 2022. Prevalence and incidence of carbapenem-resistant K. pneumoniae colonization: systematic review and meta-analysis. Syst Rev 11:240. doi:10.1186/s13643-022-02110-3 36380387 PMC9667607

[15] Chanishvili N. 2012. Phage therapy—history from twort and d'herelle through soviet experience to current approaches. Adv Virus Res 83:3–40. doi:10.1016/B978-0-12-394438-2.00001-3 22748807

[16] Hesse S, Malachowa N, Porter AR, Freedman B, Kobayashi SD, Gardner DJ, Scott DP, Adhya S, DeLeo FR. 2021. Bacteriophage treatment rescues mice infected with multidrug-resistant Klebsiella pneumoniae ST258. mBio 12:e00034-21. doi:10.1128/mBio.00034-21 33622728 PMC8545083

[17] Henrici De Angelis L, Poerio N, Di Pilato V, De Santis F, Antonelli A, Thaller MC, Fraziano M, Rossolini GM, D’Andrea MM. 2021. Phage resistance is associated with decreased virulence in KPC-producing Klebsiella pneumoniae of the clonal group 258 clade II lineage. Microorganisms 9:762. doi:10.3390/microorganisms9040762 33917365 PMC8067426

[18] Shi Y, Peng Y, Zhang Y, Chen Y, Zhang C, Luo X, Chen Y, Yuan Z, Chen J, Gong Y. 2021. Safety and efficacy of a phage, kpssk3, in an in vivo model of carbapenem-resistant hypermucoviscous Klebsiella pneumoniae bacteremia. Front Microbiol 12:613356. doi:10.3389/fmicb.2021.613356 34093455 PMC8175031

[19] Rossi M, Chatenoud L, Gona F, Sala I, Nattino G, D’Antonio A, Castelli D, Itri T, Morelli P, Bigoni S, et al.. 2021. Characteristics and clinical implications of carbapenemase-producing Klebsiella pneumoniae colonization and infection, Italy. Emerg Infect Dis 27:1416–1426. doi:10.3201/eid2705.203662 33900910 PMC8084501

[20] Lehman HK, Segal BH. 2020. The role of neutrophils in host defense and disease. J Allergy Clin Immunol 145:1535–1544. doi:10.1016/j.jaci.2020.02.038 32283205 PMC8912989

[21] Zagaliotis P, Michalik-Provasek J, Gill JJ, Walsh TJ. 2022. Therapeutic bacteriophages for Gram-negative bacterial infections in animals and humans. Pathog Immun 7:1–45. doi:10.20411/pai.v7i2.516 PMC9596135 36320594

[22] Zagaliotis P, Michalik-Provasek J, Mavridou E, Naing E, Vizirianakis IS, Chatzidimitriou D, Gill JJ, Walsh TJ. 2018. Bacteriophage treatment against carbapenem-resistant Klebsiella pneumoniae (KPC) in a neutropenic murine model of gastrointestinal translocation and renal infection. bioRxiv. doi:10.1101/2024.06.21.600121 PMC11823626 39704532

[23] Diallo K, Dublanchet A. 2022. Benefits of combined phage-antibiotic therapy for the control of antibiotic-resistant bacteria: a literature review. Antibiotics (Basel) 11:839. doi:10.3390/antibiotics11070839 35884092 PMC9311689

[24] Łusiak-Szelachowska M, Międzybrodzki R, Drulis-Kawa Z, Cater K, Knežević P, Winogradow C, Amaro K, Jończyk-Matysiak E, Weber-Dąbrowska B, Rękas J, Górski A. 2022. Bacteriophages and antibiotic interactions in clinical practice: what we have learned so far. J Biomed Sci 29:23. doi:10.1186/s12929-022-00806-1 35354477 PMC8969238

[25] Chan BK, Sistrom M, Wertz JE, Kortright KE, Narayan D, Turner PE. 2016. Phage selection restores antibiotic sensitivity in MDR Pseudomonas aeruginosa. Sci Rep 6:26717. doi:10.1038/srep26717 27225966 PMC4880932

[26] Xuan G, Lin H, Kong J, Wang J. 2022. Phage resistance evolution induces the sensitivity of specific antibiotics in Pseudomonas aeruginosa PAO1. Microbiol Spectr 10:e0135622. doi:10.1128/spectrum.01356-22 35972274 PMC9603957

[27] Hesse S, Rajaure M, Wall E, Johnson J, Bliskovsky V, Gottesman S, Adhya S. 2020. Phage resistance in multidrug-resistant Klebsiella pneumoniae ST258 evolves via diverse mutations that culminate in impaired adsorption. mBio 11:e02530-19. doi:10.1128/mBio.02530-19 31992617 PMC6989104

[28] Fontana C, Favaro M, Campogiani L, Malagnino V, Minelli S, Bossa MC, Altieri A, Andreoni M, Sarmati L. 2021. Ceftazidime/avibactam-resistant Klebsiella pneumoniae subsp. pneumoniae isolates in a tertiary Italian hospital: identification of a new mutation of the carbapenemase type 3 (KPC-3) gene conferring ceftazidime/avibactam resistance. Microorganisms 9:2356. doi:10.3390/microorganisms9112356 34835481 PMC8624296

[29] Räisänen K, Koivula I, Ilmavirta H, Puranen S, Kallonen T, Lyytikäinen O, Jalava J. 2019. Emergence of ceftazidime-avibactam-resistant Klebsiella pneumoniae during treatment, Finland, December 2018. Euro Surveill 24:1900256. doi:10.2807/1560-7917.ES.2019.24.19.1900256 31088601 PMC6518965

[30] Egido JE, Costa AR, Aparicio-Maldonado C, Haas P-J, Brouns SJJ. 2022. Mechanisms and clinical importance of bacteriophage resistance. FEMS Microbiol Rev 46:fuab048. doi:10.1093/femsre/fuab048 34558600 PMC8829019

[31] McGee LW, Barhoush Y, Shima R, Hennessy M. 2023. Phage-resistant mutations impact bacteria susceptibility to future phage infections and antibiotic response. Ecol Evol 13:e9712. doi:10.1002/ece3.9712 36620417 PMC9817185

[32] Hasan M, Ahn J. 2022. Evolutionary dynamics between phages and bacteria as a possible approach for designing effective phage therapies against antibiotic-resistant bacteria. Antibiotics (Basel) 11:915. doi:10.3390/antibiotics11070915 35884169 PMC9311878

[33] Opoku-Temeng C, Malachowa N, Kobayashi SD, DeLeo FR. 2022. Innate host defense against Klebsiella pneumoniae and the outlook for development of immunotherapies. J Innate Immun 14:167–181. doi:10.1159/000518679 34628410 PMC9149408

[34] Opstrup KV, Bennike TB, Christiansen G, Birkelund S. 2023. Complement killing of clinical Klebsiella pneumoniae isolates is serum concentration dependent. Microbes Infect 25:105074. doi:10.1016/j.micinf.2022.105074 36336240

[35] Jensen TS, Opstrup KV, Christiansen G, Rasmussen PV, Thomsen ME, Justesen DL, Schønheyder HC, Lausen M, Birkelund S. 2020. Complement mediated Klebsiella pneumoniae capsule changes. Microbes Infect 22:19–30. doi:10.1016/j.micinf.2019.08.003 31473336

[36] Abreu AG, Barbosa AS. 2017. How Escherichia coli circumvent complement-mediated killing. Front Immunol 8:452. doi:10.3389/fimmu.2017.00452 28473832 PMC5397495

[37] Hyams C, Camberlein E, Cohen JM, Bax K, Brown JS. 2010. The Streptococcus pneumoniae capsule inhibits complement activity and neutrophil phagocytosis by multiple mechanisms. Infect Immun 78:704–715. doi:10.1128/IAI.00881-09 19948837 PMC2812187

[38] Petraitiene R, Petraitis V, Kavaliauskas P, Maung BBW, Khan F, Naing E, Aung T, Zigmantaite V, Grigaleviciute R, Kucinskas A, Stakauskas R, Georgiades BN, Mazur CA, Hayden JA, Satlin MJ, Walsh TJ. 2020. Pharmacokinetics and efficacy of ceftazidime-avibactam in the treatment of experimental pneumonia caused by Klebsiella pneumoniae carbapenemase-producing K. pneumoniae in persistently neutropenic rabbits. Antimicrob Agents Chemother 64:e02157-19. doi:10.1128/AAC.02157-19 32015048 PMC7179283

[39] Petraitis V, Petraitiene R, Satlin M, Maung BBW, Khan F, Mazur C, Georgiades B, Hayden JA, Walsh TJ. 2017. Pharmacokinetics of ceftazidime/avibactam following intravenous administration in rabbits: developing the preclinical foundation for treatment of KPC-kp pneumonia in immunocompromised patients. Open Forum Infect Dis 4:S290–S291. doi:10.1093/ofid/ofx163.660

[40] Michalik-Provasek J, Lessor L, Mavridou E, Zagaliotis P, Walsh T, Gill J. 2020. Developing and characterizing bacteriophage for therapeutic use against carbapenem-resistant Enterobacteriaceae in a multiple drug resistant Klebsiella pneumoniae model. FASEB J 34:1–1. doi:10.1096/fasebj.2020.34.s1.00500

[41] Anderson B, Rashid MH, Carter C, Pasternack G, Rajanna C, Revazishvili T, Dean T, Senecal A, Sulakvelidze A. 2011. Enumeration of bacteriophage particles: comparative analysis of the traditional plaque assay and real-time QPCR- and nanosight-based assays. Bacteriophage 1:86–93. doi:10.4161/bact.1.2.15456 22334864 PMC3278645

[42] Le T, Nang SC, Zhao J, Yu HH, Li J, Gill JJ, Liu M, Aslam S. 2023. Therapeutic potential of intravenous phage as standalone therapy for recurrent drug-resistant urinary tract infections. Antimicrob Agents Chemother 67:e0003723. doi:10.1128/aac.00037-23 36975787 PMC10112157

[43] Thomas P, Sekhar AC, Upreti R, Mujawar MM, Pasha SS. 2015. Optimization of single plate-serial dilution spotting (SP-SDS) with sample anchoring as an assured method for bacterial and yeast cfu enumeration and single colony isolation from diverse samples. Biotechnol Rep (Amst) 8:45–55. doi:10.1016/j.btre.2015.08.003 28352572 PMC4980700

